# Physical Activity in Work and Leisure Time during Pregnancy, and Its Influence on Maternal Health and Perinatal Outcomes

**DOI:** 10.3390/jcm13030723

**Published:** 2024-01-26

**Authors:** Ernesto González-Cazorla, Ana Pilar Brenes-Romero, María José Sánchez-Gómez, Elena Estévez-Ruiz, Antonio Díaz-Enjuto, Ana Cantón-Cisneros, Daniel Lubián-López, Juan Mozas-Moreno, Ernesto S González-Mesa

**Affiliations:** 1 Department of Physiotherapy, Health Sciences School, Malaga University, 29010 Málaga, Spain; 2 Department of Obstetrics and Gynecology, Regional University Hospital of Málaga, 29001 Málaga, Spain; 3 Department of Obstetrics and Gynecology, Viamed Bahía de Cádiz Hospital, 11130 Cádiz, Spain; 4 Department of Obstetrics and Gynecology, University of Granada, 18016 Granada, Spain; 5 Obstetrics and Gynecology Service, Virgen de las Nieves University Hospital, 18014 Granada, Spain; 6 Consortium for Biomedical Research in Epidemiology & Public Health (CIBER Epidemiología y Salud Pública-CIBERESP), 28029 Madrid, Spain; 7 Biohealth Research Institute (Instituto de Investigación Biosanitaria Ibs. GRANADA), 18014 Granada, Spain; 8 Surgical Specialties, Biochemistry and Immunology Department, Málaga University, 29071 Málaga, Spain; 9 Research Group in Maternal-Fetal Medicine, Epigenetics, Women’s Diseases and Reproductive Health, Biomedical Research Institute of Malaga (IBIMA-Plataforma Bionand), 29071 Málaga, Spain

**Keywords:** physical activity, sedentary lifestyle, pregnancy, perinatal outcomes, leisure time, worktime

## Abstract

Background: Physical inactivity during pregnancy has been shown to be linked to an increased risk of complications. However, during pregnancy, doubts arise about what type, intensity and frequency of physical activity are most recommended. Objective: Our main objective was to know the level of physical activity (PA) and sedentary lifestyle in a representative sample of pregnant women in Málaga, one of the most populated cities in Spain. Also, we aimed to find out the effects of PA on obstetric and perinatal outcomes and on the mental health of pregnant women, differentiated according to PA intensity and domain. Methods: Five hundred and forty full-term pregnant women who had their obstetric checks in the maternity ward of the Regional University Hospital of Málaga were recruited through consecutive sampling. Participants answered a questionnaire that included the WHO Global Physical Activity Questionnaire (GPAQ), the Edinburgh Depression Scale (EDS), the Generalized Anxiety Disorder Scale (GAD-7) and some other sociodemographic and health-related questions. Subsequently, information about perinatal outcomes was obtained after birth. Results: Only 50.8% of women followed the WHO recommendations on activity. We found a high proportion of obese pregnant women and a direct effect of a sedentary lifestyle on the rate of cesarean sections and vulvovaginal tears in spontaneous births, as well as on the mental health of future mothers. Women’s age, the number of children, BMI at the beginning of pregnancy and leisure time physical activity (LTPA) explained anxiety scores, and age, LTPA, BMI at the end of pregnancy and intense work-related physical activity (WTPA) predicted depression scores. Conclusions: LTPA improves obstetric outcomes, helping to reduce the rate of cesarean sections and vulvovaginal tears, as well as reducing prenatal anxiety and depression.

## 1. Introduction

The sedentary lifestyle refers to activities carried out while awake in a sitting, reclining or lying position that involve very low energy expenditure (under 1.5 METs) [1]. It involves not only a decrease in physical activity (PA) but also postural patterns, leading to a reduced daily metabolic rate [2]. This may occur in different situations such as work, school environment, home, free time, or commuting, frequently associated with other harmful conducts, such as smoking or increased caloric intake [3]. According to the 2020 European Health Survey, 32.3% of men and 40.3% of women spend their free time completely sedentary [4]. The female population is being progressively affected by the effects of a sedentary lifestyle [5], and among them, pregnant women. Pregnancy is a period of significant anatomical and physiological changes that require continuous adaptation [6]. Likewise, it is a stage of greater vulnerability to mental disorders in women [7], with between 7% and 15% being affected [8].

The regular practice of PA is widely recognized for its health benefits, and specifically during pregnancy promotes maternal and neonatal health, reducing the risk of preterm birth, diabetes mellitus, excessive gestational weight gain and low Apgar test scores [9,10] and improving mental-health-related quality of life [11]. In the last decade, there has been a proliferation of public health guidelines on PA during pregnancy, most of which support moderate-intensity PA/exercise during pregnancy with specific frequency and duration/time recommendations [10]. Although high-intensity activity is generally deemed safe for most women, current guidelines suggest that moderate-intensity physical activity is preferable during pregnancy [12]. These guidelines also emphasize that intensity should be determined through assessments of subjective effort [13]. Nevertheless, in both medical and non-medical environments, there are often uncertainties and fears regarding the sort, intensity and frequency of physical activity or exercise that would be most advisable for individual women. These worries lead to doubt among pregnant individuals, resulting in elevated levels of physical inactivity, especially in the third trimester [14,15].

Physical inactivity during pregnancy is linked to an increased risk of complications, such as an increased likelihood of infant admission to neonatal intensive care units, preterm birth, intrauterine growth restriction, and increased caesarean section rates [16]. It is known that inactivity produces fluid shifts to accommodate perfusion to all organ systems, with specifically detrimental effects on uterine blood flow [17], and significant physiological changes occur after only a few days of immobility [18,19].

Nevertheless, it is important to note that not all physical activity (PA) may be appropriate, depending on factors such as its intensity or domain [20]. Hence, engaging in some physical activity linked to work (WTPA) and engaging in excessive physical activity during leisure time might potentially pose a risk to the advancement of pregnancy, given certain circumstances [21].

This study has two primary objectives. Initially, our objective was to ascertain the extent of physical activity and sedentary behavior among a representative sample of pregnant women in Málaga, a highly populated city in Spain. Secondly, our objective was to determine the impact of physical activity (PA) on obstetric and perinatal outcomes, as well as the mental well-being of pregnant women. We wanted to differentiate the effects based on the intensity and kind of PA.

## 2. Methods

This research responds to a descriptive design with a cross-sectional evaluation of the level of physical activity during pregnancy in full-term pregnant women to study its relationship with certain health variables, such as obstetric and perinatal outcomes and the mental health of future mothers.

### 2.1. Procedures

Between March and June 2023, a total of 540 full-term pregnant women who had their obstetric checks in the maternity ward of the Regional University Hospital of Málaga were recruited through consecutive sampling. This hospital belongs to the Andalusian Public Health System and attends an average of 5000 births per year, being the reference center for obstetric and perinatal care in the whole province of Málaga (Spain). This is a representative sample of all pregnant women treated in the area, for a confidence level higher than 95% and a margin of error of less than 5%.

Eligibility criteria based on previously established inclusion and exclusion criteria were followed. All pregnant women who did not have adequate knowledge of the Spanish language, twin pregnancies and those who either had severe functional limitations for activities of daily living (severely handicapped) or had a medical prescription for a reduction of PA during pregnancy (e.g., cervical insufficiency or preterm premature rupture of fetal membranes) were excluded from the study. No pregnant woman was excluded from the study based on previous or current BMI.

Physiotherapists of the research team and obstetricians and midwives of the staff of the regional university participated in the recruitment process. An information sheet for pregnant women and an informed consent document were prepared, which were available on the Internet through a QR code. The pregnant women used the QR code to access the questionnaire (see supplementary file). The questionnaire included sociodemographic questions, such as age, profession, employment status, district of residence and number of children, as well as a series of questions referring to the evolution of the pregnancy, such as sports habits, initial and current weight and the existence of medical or obstetric pathology. The level of physical activity was assessed using the WHO Global Physical Activity Questionnaire [22] (GPAQ), the level of depression was assessed using the Edinburgh Depression Scale [23] (EDS) and the assessment of the level of anxiety was performed using the Generalized Anxiety Disorder Scale (GAD-7) [24].

Once the pregnant women gave birth, the medical records were reviewed to blindly collect data on the type of delivery, weight and Apgar test scores of the newborns, as well as possible episiotomy, vulvovaginal tears or other complications related to childbirth.

The study was authorized by the research ethics committee of the province of Málaga. All participants expressed their consent to participate in the study by signing the corresponding document. The research was conducted following the ethical criteria of the Declaration of Helsinki, preserving the rights and confidentiality of all patients.

### 2.2. Instruments

To assess the level of physical activity, we have used the WHO Global Questionnaire on Physical Activity [22] (GPAQ). This questionnaire has been used in more than 100 countries and is available in Spanish within the WHO STEPwise tool for surveillance, monitoring and reporting of non-communicable diseases [25]. It is a subjective measure based on the perceptions, memory and judgment of the participants. The intensity level of PA is classified as moderate or intense in the dimensions of work and leisure time and only as moderate for the displacement dimension. The questionnaire also includes the report of the participant’s sedentary behavior. Each subject’s information should be based on a typical day of a typical week. With the data provided in the questionnaire, the level of physical activity is calculated using the MET. METs express the relationship between one’s working metabolic rate and resting metabolic rate. A MET is defined as the energy cost of sitting quietly and is equivalent to a caloric intake of 1 kcal/kg/h. For the analysis of the data included in the GPAQ, it is estimated that the caloric consumption of a moderately or intensely active person is four or eight times higher, respectively, than when at rest, sitting in silence [26]. WHO recommendations on physical activity have been used for the GPAQ calculations. For adults in general, over the course of a week, including activity for work, during transport and leisure, the WHO recommends at least 150 min per week for moderate activity, or 75 for intense activity, or an equivalent combination of moderate- and intense-intensity physical exercise reaching at least 600 MET-minutes. For pregnant women, the recommendation is to do at least 150 min of moderate-intensity aerobic physical activity throughout the week, incorporating a variety of aerobic and muscle-strengthening activities and limiting the amount of time spent being sedentary. They should replace sedentary time with physical activity of any intensity [27]

We used the Edinburgh Depression Scale [23] (EDS) to assess the level of perinatal depression in participants. EDS is a self-administered questionnaire, initially developed for the detection of postpartum depressive symptoms. More recently, EDS has been used to detect depression during pregnancy [28]. It consists of 10 multiple-choice questions, with 4 alternatives each, with scores from 0 to 3 depending on the increasing severity of symptoms, so that the range of the final score is 0–30. It has been validated in a large number of languages and has been used in different countries, including Spain [29]. Several authors suggest, for the English version, different cut-off points for the diagnosis of depression in the antepartum and postpartum periods [30]. For the diagnosis of puerperal depression, scores equal to or greater than 13 are considered, and for the diagnosis of antenatal depression the recommended cut-off point is 15. However, validation studies of the Spanish version carried out in pregnant women suggest a cut-off point of 13 for the diagnosis of antenatal depression [31]. In any case, a final score higher than 10 or a non-zero answer to the question that refers to the existence of self-injurious thoughts makes the existence of depression probable, making treatment by mental health professionals necessary.

To assess the anxiety of the participants, we used the Generalized Anxiety Disorder Scale [24] (GAD-7). It is a Likert-type scale that consists of 7 questions with four possible options that score between 0 and 3, so the scores of the scale vary between 0 and 21. Scores of less than 5 are interpreted as the absence of anxiety and values of 5 to 9, 10 to 14 or 15 to 21 as mild, moderate or severe anxiety, respectively [24]. The scale has been validated in Spanish [32], showing adequate psychometric values, so that a cut-off point of 10 shows a sensitivity of 83.4% and a specificity of 96% for the diagnosis of generalized anxiety disorder.

### 2.3. Statistics

We performed a first analysis of the frequency distribution of the independent variables. To determine the level of activity of pregnant women, the criteria defined by the WHO were used, both in minutes and in calculated METs. For bivariate analyses, we used the independent sample *t*-test to compare mean values in two groups of women; when the number of groups was greater than 2, single-factor ANOVA was used. Homoscedasticity conditions were assessed using the Levene test. Post hoc analyses were performed using the Tukey test. To determine the prevalence of depression and anxiety in the sample, validated cut-off points were used to calculate the global scores of the different scales. Subsequently, after verifying that the scores of global anxiety and trait and state anxiety were normally distributed using the Shapiro–Wilk test, a bivariate analysis was performed to identify associations between variables To analyze the relationship of global scores of anxiety and depression with other quantitative variables (normally distributed), such as age, minutes of activity, total METs or weight of pregnant women, the Pearson correlation coefficient was used, calculating its level of significance. When we could not confirm the normality of the distributions, we used the non-parametric Mann–Whitney or Kruskal–Wallis tests, according to the number of categories of the variables. A *p*-value less than or equal to 0.05 was considered as statistically significant. Participants’ BMI was classified according to WHO categories (i.e., underweight, below 18.5 kg/m^2^; normal weight, 18.5–24.9 kg/m^2^; pre-obesity, 25.0–29.9 kg/m^2^; obesity class I, 30.0–34.9 kg/m^2^; obesity class II, 35.0–39.9 kg/m^2^; obesity class III, above 40 kg/m^2^) [33].

To explain some perinatal outcomes such as cesarean birth rate and the characteristics of anxiety state and depression scores, we used multiple linear regression based on the independent sociodemographic, activity, emotional and health variables considered. The collinearity between the factors was analyzed to avoid including correlated variables in the model. The models were constructed by backward step regression, finally including the variables that proved to be significantly associated in the previous bivariate analysis.

### 2.4. Population

Of the 540 patients recruited, 42 were excluded from the analyses due to various reasons. Most of the excluded participants had not completed the activity questionnaire (n = 38) and in four cases consent was revoked. Finally, the sample consisted of 498 pregnant women who were in the third trimester of pregnancy. This sample continued to be representative of the population of pregnant women in our area with the same level of confidence. The mean age of pregnant women was 32.41 years (SD 5.9), and the mean gestational age was 38.1 weeks (SD 1.3). The main sociodemographic characteristics are shown in Table 1 and Table 2.

## 3. Results

### 3.1. Physical Activity Results

The results of the GPAQ are summarized in Table 3. Only 50.8% of women followed the WHO recommendations on activity, and a total of 230 (46.1%) did not engage in LTPA (intense or mild). The proportion of active women was 65% among university-educated women, a higher proportion (Chi sq 46.7, 3 df, *p* < 0.001) than that found in participants with primary (26%) or higher (47.8%) education.

Women who met the WHO recommendations in terms of activity were less obese at the end of pregnancy (39.7%), compared to 60.3% in the case of sedentary women (Chi sq 13.8, 1 df, *p* < 0.001).

BMI values at the beginning and at the end of the pregnancy showed a Pearson’s correlation coefficient of 0.93 (*p* < 0.001). Table 4 shows the correlation coefficients of time spent on each of the activities and BMI.

Academic training significantly influenced the intense LTPA (Kruskal–Wallis st = 25.0; *p* < 0.001), as participants with higher education spent more time on this type of activity (20.32 min, SD 80.7) than those who only had primary education (17.4 min, SD 93.5).

Participants with baseline obesity (BMI above 30) maintained significantly lower overall LTPA, primarily at the expense of intense activity on which they spent 4.0 min per week on average (SD 31.8) compared to participants with BMI below 30 who spent on average 35.1 min per week (SD 96.22) (t = 5.2, 454 DF, *p* < 0.0001). Also, participants with BMI greater than 30 at the end of pregnancy showed a decrease in intense LTPA (16.1 min [SD 77.1] vs. 36.9 [SD 5.5], t = 2.63, 449 df, *p* < 0.009), and moderate LTPA (113.2 min [SD 227.4] vs. 168.43 [309.27], t = 2.1, 487 df, *p* < 0.036.

Regarding WTPA, we have observed significant differences in the performance of intense activity according to the academic level of the participants (Kruskal–Wallis st = 19.92; *p* < 0.001). Pregnant women with university studies reported a lower weekly frequency of this type of activity (48.2 min, SD 205.5) compared to pregnant women with primary education (341.3 min, SD 809.9). These differences were not observed for moderate WTPA.

Also, housewives reported less intense overall PA, not only referring to work activity (68 min per week on average, [SD 402.7] in the case of housewives compared to 176.6 [SD 424.6] in the case of external workers) but also in leisure time (t = 2.25; 307 df; *p* = 0.025).

With reference to moderate WTPA, we have observed that participants in a situation of sick leave reported a greater moderate weekly physical activity than the rest (sick leave, 569.8 min [SD 707.5]; housewives, 212.0 min [SD 518.3]; employed, 446.4 min [SD 739.6]; self-employed, 395.2 min [SD 518.2]).

Interestingly, smokers spent more time per week on moderate PA (437 min, SD 747.5) at work than non-smokers (204.3, SD 489.9, t = 4.06, 496 df, *p* = 0.001). However, smoking participants carried out less PA during commuting (35.4 min [28.5] vs. 41.4 min [26.9]; t = 2.138; 496 df; *p* = 0.034).

### 3.2. Obstetric and Perinatal Outcomes

Of the recruited women, we had information on the delivery of 373 participants who gave birth in the maternity ward of the hospital. The main obstetric and perinatal outcomes are shown in Table 5. We observed an overall cesarean section rate of 28.1%, with the mean age of pregnant women significantly higher in the cesarean section group (33.1 years [SD 5.7] versus 31.8 [SD 5.7] in the vaginal delivery group, t 2.15, 371 dof, *p* < 0.032). Participants who carried out moderate LTPA had a lower proportion of cesarean deliveries, specifically, the cesarean section rate was 30.3% among pregnant women who were active compared to 40.9% in those who were not (Chi sq 3.92, 1 dof, *p* < 0.03).

We have also observed significant differences in the type of delivery according to BMI at the beginning (F 4.43, 2 df, *p* < 0.013) and at the end of pregnancy (F 7.9, 2 df, *p* < 0.001), where women who required cesarean section were those with higher BMI (mean BMI 26.6 at the beginning of pregnancy [SD 5.0], and BMI 30.7 at the end of gestation [SD 5.0]), when compared with those who had a vaginal delivery (BMI 25.1 [SD 4.7] in the first weeks of gestation, and BMI 28.6 [SD 4.4] at term). Specifically, women with obesity criteria at the beginning of pregnancy had a cesarean section rate of 48.1% compared to 34.5% of the rest of the participants (Chi sq 4.9, 1 dof, *p* < 0.19). Similarly, pregnant women with obesity at the end of pregnancy had a higher cesarean section rate of 44.1% versus 33.5% (Chi sq 4.1, 1 dof, *p* < 0.027). The magnitude of weight gain during pregnancy did not produce significant differences in the type of delivery.

BMI at the beginning of pregnancy was associated with the development of pathological conditions during pregnancy, mainly diabetes and hypertension (t = 3.2, 495 df, *p* < 0.001), as patients with diabetes or hypertension showed BMI at the beginning of pregnancy of 27.32, compared to 25.2 in participants without pathology. Gestational diabetes was associated with obesity in 53.3% of cases, and hypertension in 60% was significantly associated with obesity in early pregnancy.

The need for episiotomy was significantly increased in primiparous women (52.8% of episiotomies, compared to 19.3 in women with previous births {Chi Sq 27.9, 2 df, *p* < 0001}). In cases of spontaneous delivery without episiotomy, we observed a lower incidence of perineal tears in the participants who carried out moderate LTPA (t = 2.2 72.3 df, *p* < 0.029). The mean duration of weekly moderate LTPA was 209.3 min [SD 321.3] in the non-tear group, versus 56.79 min for second-degree tears [SD 170.0] and 102.6 min [SD 151.8] for first-degree tears.

We have not observed significant differences in fetal weight depending on the time spent on physical activity, however, we have found a higher proportion of smokers among those who had small-for-gestational-age newborns (61% vs. 38.5% of non-smoking women). In fact, 5.8% of smokers had newborns weighing less than 2500 g, while only 1.4% of non-smokers did (Chi sq 7.75, 1 df, *p* < 0.010).

Multivariate logistic regression models indicated that women’s age, previous obesity and LTPA were predictors of the type of birth (Table 6).

### 3.3. Mental Health

Cronbach’s alpha coefficient values were 0.86 for the Edinburgh questionnaire and 0.89 for the GAD-7 questionnaire. The scores of both scales showed a significant Pearson’s correlation coefficient of 0.73, *p* < 0.0001.

The mean score for the depression questionnaire was 7.6 [SD 5.11]. The 25th, 50th and 75th percentiles were 4, 7 and 11, respectively. We found that 13.3% of participants scored above the cut-off point for antenatal depression and 5% scored greater than zero on the question about self-harm ideas. We observed a negative correlation between the EDS scores and women’s age (r Pearson −0.19, *p* < 0.0001). The mean age of women who scored above the cut-off point for depression was 30.1 (SD 6.3), while the age of those who scored below was 32.7 (SD 5.7) (t = 3.12, 495 df, *p* < 0.001). The scores were significantly higher in pregnant women with more intense WTPA, as well as in participants with higher BMI at the beginning and end of pregnancy, as shown in Table 7.

On the other hand, pregnant women with higher academic qualifications obtained significantly lower scores, as university-educated women obtained an average score of 6.69 [SD 4.7], while in pregnant women with primary and higher education the scores were 8.7 [SD 5.5] and 8.3 [SD 5.1], respectively.

Participants who scored above the cut-off point for depression spent significantly more time on intense WTPA (274.1 vs. 122.1 min in the non-depression group, Mann–Whitney’s U 2.1, *p* < 0.035), with a higher number of METs (2193.3 vs. 977.4 METs, Mann–Whitney’s U 2.1, *p* < 0.035).

The multivariate study showed that the variables that predict Edinburgh questionnaire scores were age, LTPA, BMI at the end of pregnancy and intense work-related physical activity (Table 8).

Regarding the anxiety questionnaire, the mean score obtained was 6.96 (SD 4.6), with 3, 6 and 10 being the values corresponding to the 25th, 50th and 75th percentiles. Of the participants, 25.5% scored above the cut-off point of 10, with 17.4% showing moderate anxiety and 8.03% intense anxiety. We have observed statistically significant correlations between the scores obtained in the anxiety questionnaire and BMI at the beginning of pregnancy (Pearson’s r 0.14, *p* < 0.0001), BMI at the end of gestation (Pearson’s r 0.12, *p* < 0.0001), the weekly minutes spent on intense WTPA (Pearson’s r = 0.1, *p* < 0.05), as well as with the age of the participants (Pearson’s r −0.18, *p* < 0.0001). Women with obesity in early pregnancy had a mean score of 8.1 (SD 5.4) while those who did not have obesity had a mean score of 6.7 (SD 4.3) (t = 2.34, 486 df, *p* < 0.021).

Participants with higher levels of anxiety (moderate to severe) spent significantly more time on intense WTPA (215 vs. 117 min in the group without significant anxiety, Mann–Whitney’s U 2.2, *p* < 0.02), with a higher number of METs (1720.3 vs. 939.5 METs, Mann–Whitney’s U 2.3, *p* < 0.022).

We observed lower average scores in the group of university-educated women compared to those with lower academic achievements. The mean GAD-7 score was 5.8 (SD 4.6) in the case of university women compared to 8.1 (SD 4.7) and 7.9 (SD 4.8) in the case of high school and primary education (F 9.66, 3 df, *p* < 0.0019).

The scores were also higher in participants with children, especially when they were younger than 5 years, reaching a value of 7.9 (SD 5.0) in women with at least one young child, compared to 6.4 (SD 4.3) in women without children (F 3.1, 3 df, *p* < 0.025).

Scores on the anxiety scale were higher in women without LTPA during pregnancy, with the average value of the scale being 8.03 (SD 4.8) in participants who did not perform any PA, compared to 6.4 (4.3) in those who did (F4.17, 4 df, *p* < 0.002). In fact, participants who were active according to WHO recommendations scored lower (mean dif 1.1, F 6.6, 1df, *p* < 0.01).

Pregnant women with medical complications during pregnancy also had significantly higher anxiety levels (mean difference 2.7, F 4.28, *p* < 0.03), especially asthmatic patients (mean 8.9, SD 4.7) and those with thrombophilia (mean 9.7, SD 1.5).

Participants with anxiety scores above the cut-off point carried out significantly more intense WTPA, with 215.04 min per week of intense activity (SD 584.6) in participants with moderate–severe anxiety, compared to 117.4 (SD 457.7) in the rest (Mann–Whitney U 25677.5, *p* < 0.02).

The multivariate analysis showed that women’s age, the number of children, BMI at the beginning of pregnancy and LTPA were the best predictors of anxiety scores (Table 9).

## 4. Discussion

This research aimed to assess the level of physical activity and sedentary lifestyle in the population of pregnant women from one of the most populated provinces of Spain, as well as the effects on perinatal outcomes and mental health of pregnant women.

The results showed a low rate of women who complied with WHO guidelines about PA during pregnancy, a high proportion of obese pregnant women and a direct effect of a sedentary lifestyle on the rate of cesarean sections and vulvovaginal tears in vaginal births, as well as on the mental health of future mothers.

Up to 46.1% participants did not to perform any significant physical activity during their leisure time. These data are higher than those described in the population of adult women in Andalusia [34] (over 41%) and similar to those described in previous studies conducted by other authors [35], despite 85.7% being healthy pregnant women without prescription of reduction of physical activity. We can say that our results reflect the trend observed in the general population, with a progressive increase in sedentary habits and behaviors [3], increasing the risks of the main non-communicable diseases worldwide [36,37,38] such as cardiovascular disease, hypertension, type II diabetes mellitus, colon or breast cancer, strokes or emotional disorders [39] and, specifically in pregnancy, preeclampsia, gestational diabetes, cesarean delivery and perinatal depression [40].

Technological progress makes people’s lives easier but makes them more immobile, enhances attention to screens [41,42] and reduces their physical agility and their energy expenditure. A sedentary lifestyle and obesity are closely linked [43]. In fact, 48% of the pregnant women in our study had BMI values of obesity at the beginning of pregnancy, and according to previous publications [44,45], they were the ones who most frequently suffered from hypertension and gestational diabetes. The high prevalence of sedentary lifestyle observed in pregnant women also reflects the gender gap in health that is evident in the epidemiological surveys of our environment [46]. The percentage of women who perform physical activity in their leisure time is lower than that described in men (9.8% versus 17.1%) and the proportion of women who occupy their free time sedentarily is higher (41.8% in women and 34.1% in men) [46]. These inequalities not only affect activity in leisure time but also extend to the workplace since 61.3% of women carry out habitual activities that require standing most of the time without large displacements or physical efforts, as is the case with the housewife’s domestic chores, while this only occurs in 12.5% of men. Among men, the sedentary level is equally high and progressive over the years [47], but their work activity is carried out mainly in positions that do not require standing.

Although some systematic reviews have shown that physical activity (PA) is associated with improved mental health and reduced risk of anxiety and depression [48,49,50,51], many of the studies look at total weekly physical activity, without considering the domains in which physical activity is performed. Several studies indicate differences depending on the type of activity carried out, especially concerning mental health [52,53]. WTPA, whether moderate or intense, does not have the same benefits as LTPA [49,54]. While physical activity performed in leisure time is associated with some intrinsic motivation and feelings of enjoyment, autonomy and self-efficacy, work-related activity is usually performed against the clock and under pressure regarding the result [52]. In our study, we assessed the level of physical activity during pregnancy using a tool that distinguishes between intense and moderate physical activity levels referring to work activity and leisure time. The observed benefits of physical activity in our study refer to LTPA, and when the time dedicated to work activity was included in the analyses, the benefits disappeared. In addition, participants who spent more time on WTPA were more frequent smokers, so they were exposed to additional risk factors. In our study, pregnant women who devoted more time to intense WTPA were those with higher levels of anxiety and depression.

On the other hand, while total PA is associated in multiple studies with better obstetric and perinatal outcomes [55,56,57], WTPA has been shown in others as a detrimental element that increases the risk of complications such as severe preeclampsia [58].

Our results did not show an increase in obstetric pathology or worse neonatal outcomes related to WTPA. There was no relationship between METs and neonatal weight. On the contrary, we have found benefits in pregnant women who have maintained a level of activity during pregnancy in line with WHO recommendations [27], with a lower rate of cesarean sections and vaginal tears in pregnant women who carry out moderate LTPA.

Also, the influence of the educational level of the participants reflects another type of social inequality, since, as previously reported [59,60], pregnant women with higher academic achievements (university students) were more active and, therefore, less exposed to the risks derived from sedentary behavior.

One of the main barriers that hinders physical activity during pregnancy is the lack of knowledge about the benefits for the mental health of pregnant women and for obstetric and neonatal outcomes [61,62]. Also, a higher level of studies allows access to higher-quality jobs that are compatible with LTPA. In any case, although personal factors may have more weight than environmental factors in the high prevalence of a sedentary lifestyle [63], when the conditioning factors are analyzed from a gender perspective, the contextual factors of the place of residence are also important in women [64].

To meet the objective of reducing sedentary lifestyle during pregnancy, it is necessary to implement general measures to enhance the possibilities of carrying out physical exercise but also personalized measures, with prescription of physical activity at flexible times and places, supported by professionals specialized in PA, reinforcing education and motivation [65,66,67,68]. Personalized physical activity may be an advantageous approach to effectively help pregnant women meet current physical activity recommendations.

## 5. Strengths and Limitations

In this research, we did not use any objective measure for PA. All data were self-reported, using a validated questionnaire. The use of self-reported validated questionnaires lowers the costs, simplifies the development of any study and facilitates its implementation [69]. However, self-reporting has inherent limitations, as it is prone to information, memory and classification biases. Therefore, the information collected in this work should be evaluated with this perspective. During the recruitment period, members of the research team were responsible for motivating participants to minimize these biases. However, we contribute data regarding PA according to different domains where it is performed (WTPA and LTPA) and not only global PA.

## 6. Conclusions

According to the data presented in this research, physical activity should be actively promoted and encouraged in pregnant women since it improves obstetric outcomes, helps to reduce the rate of cesarean sections and vulvovaginal tears and has positive effects on mental health, reducing prenatal anxiety and depression. In the studied population, these benefits were observed when physical activity was carried out during leisure time, with some observed negative effects of physical activity related to work activity. Sedentary behaviors during pregnancy reflect previous factors acknowledged such as a high prevalence of obesity and overweight observed at the beginning of pregnancy.

## Figures and Tables

**Table 1:** Quantitative data.

	Mean	Range	SD
Age	32.4 years	15–51 years	5.90
Pregestational weight	68.14 kg	40–135 kg	13.91
Current weight	78.16 kg	49–145 kg	13.89
Weight gain	9.96 kg	−6.0–66.2 kg	4.98
Height	1.63 m	1.48–1.87 m	0.06
BMI in early pregnancy	25.52 kg/m^2^	14.53–45.70 kg/m^2^	5.08
BMI at term	29.27 kg/m^2^	13.2–29.27 kg/m^2^	5.03

**Table 2:** Characteristics of the population.

**Academic level (498)** No studies Compulsory education Secondary education University	2.8% (14) 20.1% (100) 31.5% (157) 45.6% (227)	**BMI in early pregnancy** Underweight Normal weight Overweight Type 1 obesity Type 2 obesity Type 3 obesity	4.6% (23) 47.4% (236) 26.7% (133) 14.3% (71) 3.8% (19) 1.2% (6)
**Employment status (498)** Sick leave Unemployed Employed Self-employed	29.5%(147) 25.9% (129) 36.1% (180) 8.4% (42)	**BMI at term** Underweight Normal weight Overweight Type 1 obesity Type 2 obesity Type 3 obesity	0.2% (1) 17.5% (87) 43.6% (217) 24.1%(120) 9.4% (47) 3.4% (17)
**Children (498)** No 1 2 3	57.6% (287) 31.9%(159) 9.0%(45) 1.4%(7)	**Sport** No Once a week 2–3 times a week 3–5 times a week > 5 times a week	32.7% (163) 12.9% (64) 37.1%(185) 14.3% (71) 3.0% (15)
**Age of children (211)** Children under 5 years old Over 5 years old Both	60.6% (128) 27.9% (59) 11.4% (24)	**Justification** Lack of time Medical indication Other reasons	26.5% (89) 5.6% (19) 31.9% (107)
**Smokers** Yes No	27.5% (137) 72.5% (361)		
**Diseases** No Hypothyroidism Diabates mellitus Asthma Thrombophilia Autoimmune pathology HTA Mental health	85.7% (427) 5.4% (27) 3.0% (15) 2.4% (12) 0.8% (4) 1.0% (5) 1.0% (5) 0.2% (1)		

**Table 3:** GPAQ Results.

Physical Activity	With Activity n (%)	No Activity n (%)	Weekly Minutes Mean (SD)	METs Mean (SD)
**Work**				
Intense	96 (19.3%)	402 (80.7%)	142.3 (494.4)	1138.6 (3955.4)
Moderate	187 (37.6%)	311 (62.4%)	268.5 (581.08)	1074.1 (2324.3)
**Leisure**				
Intense	70 (85.9%)	428 (14.1%)	28.6 (89.2)	228.8 (714.1)
Moderate	263 (52.8%)	235 (47.2%)	149.3 (282.8)	597.3 (1131.4)
**Displacement**				
Yes	369 (74.1%)	129 (25.9%)	39.8 (27.5)	159.0 (110.05)
**Total METs**				3197.9 (5813.9)

**Table 4:** Main correlations.

	IMC Baseline	IMC Current	Intense Activity Work	Moderate Activity Work	Intense Activity Leisure	Moderate Activity Leisure
Intense Activity Work	0.09 *	0.1 *	-	-	-	-
Moderate Activity Work	0.09 *	0.1 *	0.5 **	-	-	-
Intense Activity Leisure	−0.13 **	−0.14 **	−0.04	−0.00	-	-
Moderate Activity Leisure	−0.10 *	−0.1 *	0.13 **	0.11 *	0.11 *	-
Displacement	−0.05	−0.05 *	0.07	0.08	−0.0	0.2 **

* *p* < 0.05; ** *p* < 0.001.

**Table 5:** Obstetric and perinatal outcomes.

**Type of delivery (373)**	
Spontaneous	163 (43.7%)
Instrumental	70 (18.8%)
Cesarean section	140 (28.1%)
**Perineum (161)**	
Episiotomy	32 (19.9%)
Degree I tear	39 (24.2%)
Degree II tear	41 (25.5%)
Perineum intact	49 (30.4%)
**Newborn**	
Female	213 (49.2%)
Male	220 (50.8%)
Apgar 1st	Median = 9 (SD = 0.76)
Apgar 5th	Median = 10 (SD = 0.44)
Weight (gr)	Mean = 3357.1 (SD = 431.0)

**Table 6:** Logistic Regression Model. OR values for cesarean delivery.

	B	S.E.	Itself.	OR	OR 95% C.I.	OR 95% C.I.
					Lower	Upper
LTPA	0.455	0.241	0.05	1.576	0.982	2.527
Obesity First Trimester	−0.533	0.259	0.03	0.587	0.353	0.975
Age	0.04	0.02	0.05	1.04	1.001	1.084
Constant	−0.417	0.290	0.151	0.659		

**Table 7:** Physical activity and depression.

	Depression	Mean	Std. Dev	Sig. (Mann–Whitney U)
BMI at baseline (kg/m^2^)	No (n = 432)	25.27	4.88	t = 16,645.5 *p* < 0.0
	Yes (n = 66)	27.11	5.99	
BMI at term (kg/m^2^)	No (n = 432)	29.11	4.92	t = 16,549.5 *p* < 0.035
	Yes (n = 66)	30.49	5.29	
Weight at baseline (kg)	No (n = 432)	67.42	13.30	t = 16,767.5 *p* < 0.019
	Yes (n = 66)	72.82	16.74	
Term weight (kg)	No (n = 432)	77.64	13.46	t = 16,537.5 *p* < 0.036
	Yes (n = 66)	81.89	15.28	
WTPA i	No (n = 432)	122.19	452.18	t = 15,764.5 *p* < 0.035
	Yes (n = 66)	274.17	702.16	
WTPA m	No (n = 432)	261.98	571.25	ns
	Yes (n = 66)	311.33	644.84	
LTPA i	No (n = 432)	24.85	81.12	ns
	Yes (n = 66)	53.18	128.77	
LTPA m	No (n = 432)	149.14	286.15	ns
	Yes (n = 66)	150.53	262.24	
PA and	No (n = 432)	147.03	455.52	t = 16,453.5 *p* < 0.011
	Yes (n = 66)	327.35	703.72	
PA m	No (n = 432)	411.12	666.37	ns
	Yes (n = 66)	461.86	722.39	

ns, not significant.

**Table 8:** Multiple regression models for EDS scores.

Depression		Unstandardized Coefficients		Standardized Coefficients	t	Significance	R^2^
		B	Std. Error	Beta			
1	(Constant)	12.940	1.270		10.188	0.000	0.036
	Age	−0.163	0.039	−0.189	−4.233	0.000	
2	(Constant)	13.342	1.272		10.492	0.000	0.045
	Age	−0.149	0.039	−0.172	−3.848	0.000	
	LTPA	−1.283	0.487	−0.118	−2.632	0.009	
3	(Constant)	10.331	1.892		5.459	0.000	0.058
	Age	−0.144	0.039	−0.167	−3.741	0.000	
	LTPA	−1.245	0.486	−0.114	−2.561	0.011	
	Final BMI	0.097	0.045	0.095	2.142	0.033	
4	(Constant)	9.890	1.893		5.224	0.000	0.069
	Age	−0.146	0.038	−0.169	−3.792	0.000	
	LTPA	−1.391	0.488	−0.128	−2.851	0.005	
	Final BMI	0.111	0.045	0.109	2.441	0.015	
	WTPA	0.006	0.003	0.104	2.324	0.021	

**Table 9:** Multiple regression models for GAD-7 scores.

Anxiety		Unstandardized Coefficients		Standardized Coefficients	t	Significance	R^2^
		B	Std. Error	Beta			
1	(Constant)	11.471	1.157		9.914	0.000	0.029
	Age	−0.138	0.035	−0.176	−3.944	0.000	
2	(Constant)	11.503	1.142		10.071	0.000	0.054
	Age	−0.157	0.035	−0.200	−4.493	0.000	
	Number of children	1.073	0.289	0.165	3.707	0.000	
3	(Constant)	8.588	1.554		5.528	0.000	0.072
	Age	−0.153	0.035	−0.195	−4.406	0.000	
	Number of children	1.046	0.288	0.161	3.636	0.000	
	Initial BMI	0.110	0.040	0.120	2.746	0.006	
4	(Constant)	9.043	1.556		5.810	0.000	0.084
	Age	−0.139	0.035	−0.177	−3.949	0.000	
	Number of children	0.902	0.292	0.139	3.087	0.002	
	Initial BMI	0.105	0.040	0.116	2.650	0.008	
	LTPA	−1.103	0.445	−0.111	−2.477	0.014	

## Data Availability

The Dataset is available from corresponding author upon reasonable request.

## Supplementary Materials

The following supporting information can be downloaded at: https://www.mdpi.com/article/10.3390/jcm13030723/s1.

## Abbreviations

PA	physical activity
LTPA	leisure time physical activity
WTPA	work time physical activity
BMI	body mass index
SD	standard deviation
