# Supplementary material for: Physical Activity in Work and Leisure Time during Pregnancy, and Its Influence on Maternal Health and Perinatal Outcomes
Source: J Clin Med. 2024 Jan 26;13(3):723. doi: 10.3390/jcm13030723 (PMC10856528; doi:10.3390/jcm13030723)
Supplement: Supplementary file 1 [file jcm-13-00723-s001.zip › jcm-2753826-supplementary.pdf]

## Information sheet and informed consent

Today, society has given physical exercise and sport a primary role in the preservation and development of physical and mental health in human beings. Over the years, and thanks to the contribution of science, efforts have been made to promote the link between physical exercise and pregnancy as much as possible due to its immense benefits. However, the prevalence of insufficiently active women is as high as 92 per cent at the national level.

Therefore, within the line of this project is to promote physical activity during pregnancy, evaluate the level of physical activity of pregnant women in our environment in the last trimester and evaluate the influence of physical activity on obstetric and neonatal outcomes and mental health at the end of pregnancy.

Consent Code: ...............

Mrs. ............................................, with ....... years of age, and ID nº ......................,

I declare that I have read and understood the information sheet that has been given to me.
I asked the questions that arose about the project and that I have received sufficient information about it.
I understand that my participation is completely voluntary, that I can withdraw from the study at any time without having to explain the reasons and without this affecting my medical care.
I freely agree to participate in the Research Project entitled " physical activity in pregnant women in our environment and its influence on perinatal obstetric outcomes and peripartum mental health".
I have also been informed that my personal data will be protected and included in a file that must be subject to and with the guarantees of the General Data Protection Regulation (GDPR), which came into force on May 25, 2018, which means the repeal of Organic Law 15/1999, of December 13, 1999, referring to the protection of natural persons with regard to the processing of personal data

Taking this into consideration, I GIVE my CONSENT to cover the objectives specified in the project.

Signature

Málaga, date:
